# Dynamic Chloroplast Genome Rearrangement and DNA Barcoding for Three Apiaceae Species Known as the Medicinal Herb “Bang-Poong”

**DOI:** 10.3390/ijms20092196

**Published:** 2019-05-04

**Authors:** Hyun Oh Lee, Ho Jun Joh, Kyunghee Kim, Sang-Choon Lee, Nam-Hoon Kim, Jee Young Park, Hyun-Seung Park, Mi-So Park, Soonok Kim, Myounghai Kwak, Kyu-yeob Kim, Woo Kyu Lee, Tae-Jin Yang

**Affiliations:** 1 Department of Plant Science, Plant Genomics and Breeding Institute, and Research Institute for Agriculture and Life Sciences, College of Agriculture and Life Sciences, Seoul National University, Seoul 08826, Korea; dlgusdh88@snu.ac.kr (H.O.L.); zerosight@snu.ac.kr (H.J.J.); kkhpyh@hanmail.net (K.K.); nhkim@phyzen.com (N.-H.K.); jypark74@snu.ac.kr (J.Y.P.); fgenesis@snu.ac.kr (H.-S.P.); 2 Phyzen Genomics Institute, 605, Baekgoong Plaza1, Seongnam 13558, Korea; sclee0923@hanmail.com (S.-C.L.); iamparkmiso@naver.com (M.-S.P.); 3 Genetic Resources Assessment Division, National Institute of Biological Resources, Incheon 404-170, Korea; sokim90@korea.kr; 4 Plant Resources Division, National Institute of Biological Resources, Incheon 404-170, Korea; mhkwak1@korea.kr; 5 Herbal Medicine Research Division, Ministry of Food and Drug Safety, Cheongju 28159, Korea; yeop007@korea.kr; 6 Criminal Investigation Office, Ministry of Food and Drug Safety, Cheongju 28159, Korea; leewk1977@korea.kr

**Keywords:** *Ledebouriella seseloides*, *Peucedanum japonicum*, *Glehnia littoralis*, chloroplast genome, inverted repeat (IR) expansion, phylogenetic analysis, divergence time estimation

## Abstract

Three Apiaceae species *Ledebouriella seseloides*, *Peucedanum japonicum*, and *Glehnia littoralis* are used as Asian herbal medicines, with the confusingly similar common name “Bang-poong”. We characterized the complete chloroplast (cp) genomes and 45S nuclear ribosomal DNA (45S nrDNA) sequences of two accessions for each species. The complete cp genomes of *G. littoralis*, *L. seseloides*, and *P. japonicum* were 147,467, 147,830, and 164,633 bp, respectively. Compared to the other species, the *P. japonicum* cp genome had a huge inverted repeat expansion and a segmental inversion. The 45S nrDNA cistron sequences of the three species were almost identical in size and structure. Despite the structural variation in the *P. japonicum* cp genome, phylogenetic analysis revealed that *G. littoralis* diverged 5–6 million years ago (Mya), while *P. japonicum* diverged from *L. seseloides* only 2–3 Mya. Abundant copy number variations including tandem repeats, insertion/deletions, and single nucleotide polymorphisms, were found at the interspecies level. Intraspecies-level polymorphism was also found for *L. seseloides* and *G. littoralis*. We developed nine PCR barcode markers to authenticate all three species. This study characterizes the genomic differences between *L. seseloides*, *P. japonicum*, and *G. littoralis*; provides a method of species identification; and sheds light on the evolutionary history of these three species.

## 1. Introduction

Apiaceae (Umbelliferae) is one of the largest families of flowering plants. It comprises approximately 450 genera and 3700 species, which are widely distributed across the temperate zone [1,2]. This family is characterized by a simple or complex compound inflorescence, called an umbel, which resembles the ribs of an umbrella. The family includes well-known plant species used for cooking, such as carrot (*Daucus carota*), celery (*Apium graveolens*), and parsley (*Petroselinum crispum*), as well as many others used in traditional medicine in Eastern Asia [3].

The Apiaceae species *Ledebouriella seseloides* (Hoffm.) H. Wolff is known as Bang-poong in Korea, Fang-feng in China, and Bo-fu in Japan, all rendered with the same Chinese characters [3,4,5]. The dried roots and rhizomes of *L. seseloides* are used in traditional medicine to treat headaches, vertigo, and generalized aching [6]. Two other Apiaceae species, *Peucedanum japonicum* Thunb. (*n* = 11) [7] and *Glehnia littoralis* F. Schmidt ex Miq. (*n* = 11) [8], have similar common names, Sik-Bang-Poong and Hae-Bang-Poong, respectively, and are sold in traditional Korean medicine markets as alternatives to *L. seseloides* [5]. Thus, it is necessary to clearly classify each species and to develop a DNA barcoding tool to authenticate each species.

Plant genetic barcode markers are often based on chloroplast (cp) genomes and 45S nuclear ribosomal DNA (nrDNA) because these sequences are well conserved across plant species and show clear interspecies polymorphism with rare intraspecies polymorphism [9]. The cp genome is a circular molecule of 120–217 kb, which generally comprises a large single-copy (LSC) region, a small single-copy (SSC) region, and two inverted repeat (IR) regions [9,10]. The 45S nrDNA cistron unit encodes 5.8S, 18S, and 26S rRNAs, separated by internal transcribed spacer 1 (ITS1) and ITS2 [9]. To classify plants and develop molecular markers, studies have analyzed regions including intergenic spacers (IGSs) or coding sequences in the cp genome, and ITS sequences in 45S nrDNA [9,11,12]. Notably, both cp genome DNA and 45S nrDNA are highly abundant in plant tissue extracts because there are hundreds of copies of the cp genome in cytoplasmic organelles and thousands of tandem array copies of 45S nrDNA in cell nuclei [9]. 

In this study, we characterized the complete cp genomes and 45S nrDNA cistron units of three medicinal plants, *L. seseloides*, *P. japonicum*, and *G. littoralis*, using a low-coverage whole-genome sequencing (WGS) approach (dnaLCW) for highly efficient, simultaneous de novo assembly of cp and 45S nrDNAs [13]. Comparative genome analysis revealed the phylogenetic relationships between these three species and highlighted dramatic structural changes in the cp genome of *P. japonicum*. We also developed authentication markers to classify the three species. This study provides valuable genetic resources for authenticating three commercially important plant species, as well as for the taxonomical classification of Apiaceae species.

## 2. Results

### 2.1. Six Complete Cp Genomes and 45S Nrdna Sequences of Three Apiaceae Species

**Figure 1:**
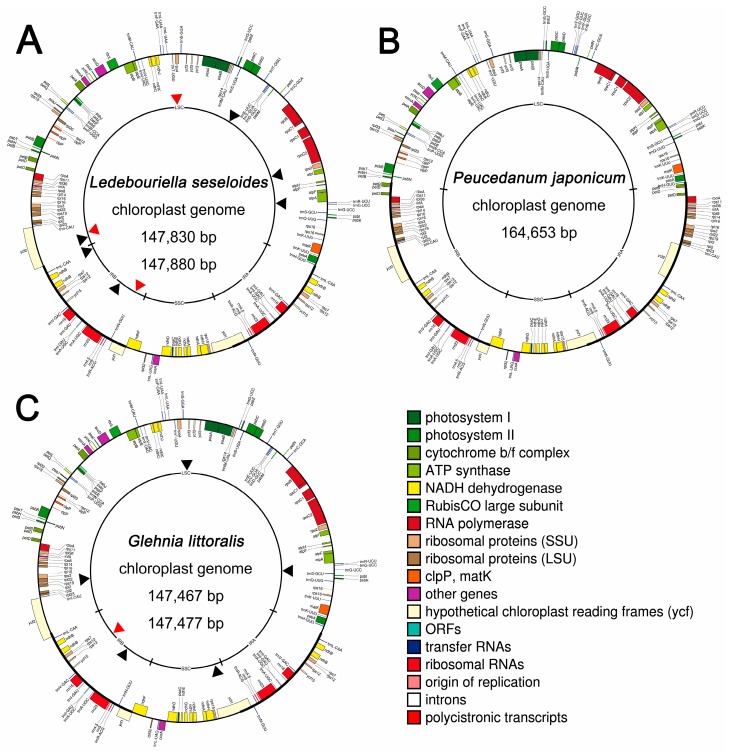
Chloroplast (cp) genome maps of *Ledebouriella seseloides* (**A**), *Peucedanum japonicum* (**B**), and *Glehnia littoralis* (**C**). Colored boxes are conserved cp genes, annotated using the DOGMA program, with manual editing based on BLAST searches. Maps were generated using OGDraw. Genes transcribed clockwise and counterclockwise are indicated on the outside and inside of the large circle, respectively. The intraspecies polymorphic sites 9, 0, and 6 are indicated by black arrowheads for indels and red arrowheads for SNPs in the inner circles of A and C.

Using two accessions each of *L. seseloides* (Ls-01 and Ls-02), *P. japonicum* (Pj-01 and Pj-02), and *G. littoralis* (Gl-01 and Gl-02), 1.06–5.18 Gb of raw WGS data were generated (Table 1). Complete sequences of the cp genomes and 45S nrDNA for each accession were successfully assembled using the dnaLCW method. Lengths of the complete cp genomes for each accession were as follows: Ls-01, 147,880 bp; Ls-02, 147,830 bp (Table 1, Figure 1A); Pj-01 and Pj-02, both 164,653 bp (Table 1, Figure 1B); Gl-01 147,467 bp; and Gl-02, 147,477 bp (Table 1, Figure 1C). The gene contents of each cp genome were identical among the six accessions. A total of 120 genes, including 85 protein-coding genes, 31 transfer RNA (tRNA) genes, and four ribosomal RNA (rRNA) genes, were commonly annotated in all cp genomes (Supplementary Table S1).

For each of the three plant species, 45S nrDNA sequences were assembled into single contigs, including the complete 45S cistron unit (hereafter known as the 45S nrDNA unit) (Table 1). As described in a previous study [14], it was not possible to assemble complete IGSs because of gaps at GC-rich regions within them, so only 45S nrDNA unit sequences were analyzed further in this study. The 45S nrDNA units were identical between each of the accessions of a single species, except for a single heterogeneous nucleotide found in *L. seseloides* (Table S2). Lengths of the 45S nrDNA units were identical between *L. seseloides* and *P. japonicum* (5185 bp), which was 3 bp longer than *G. littoralis* (5812 bp) (Table 1). 

### 2.2. Identification of Intraspecies Variations within Three Apiaceae Cp Genomes 

The cp genome variations are rarely identified at the intraspecies level. Intraspecies sequence variation was investigated by comparing the cp genome sequences of the two accessions of each species (*L. seseloides*, *P. japonicum*, and *G. littoralis*). Nine sequence variations, including six indels and three SNPs, were found between the two accessions of *L. seseloides* (Figure 1A, Table S3), and six variations, including five indels and a single SNP, were found between the two *G. littoralis* accessions (Figure 1B, Table S4). No intraspecies sequence variation was present between the two *P. japonicum* accessions (Figure 1C).

### 2.3. IR Expansion and Structural Variations among Three Apiaceae Species 

**Figure 2:**
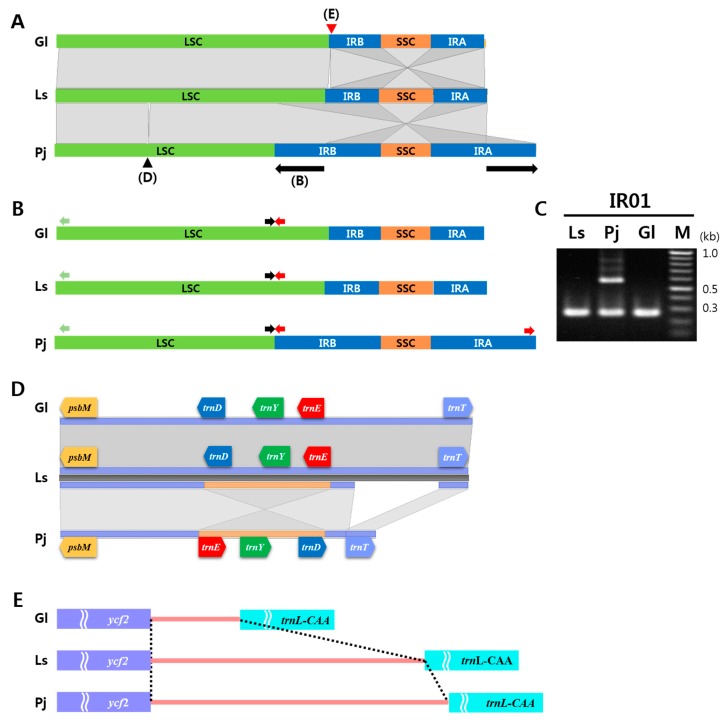
Comparative analysis of chloroplast (cp) genomes structure between the three Apiaceae species. (**A**) Blast-Z alignment of three species. Expanded inverted repeat (IR) regions (IRA and IRB) found in *Peucedanum japonicum* are indicated by arrows. (**B**) Locations of the three primers used to detect IR expansion in the cp genome sequences of three Apiaceae species. Black and red arrows indicate forward and reverse primers for control amplicons amplified in all three species, while the green arrows indicate primers specific to the left proximal junction region of the large single copy (LSC). Note that the site for the control reverse primer (red) is also present in the IRA region of the *P. japonicum* cp genome, resulting in amplification of one additional DNA fragments. (**C**) PCR results to validate the IR junction. A DNA fragment of about 0.6 kb in length was only amplified in *P. japonicum*, while a DNA fragment of about 0.3 kb amplified by control primers was detected in all three Apiaceae species. PCR amplicons were analyzed by 1.3% agarose gel electrophoresis. (**D**) Gene inversion found in *P. japonicum*. A 625-bp region, including *trn*D-GUC, *trn*Y-GUA, and *trn*E-UUC, was inverted in *P. japonicum* (indicated by black arrowhead in A), unlike in *Glehnia littoralis* and *Ledebouriella seseloides*. (**E**) Variable intergenic region between the three species. The *ycf*2 ~ *trn*L-CAA intergenic region (indicated by red arrowhead in A) in *G. littoralis* (391 bp) was much shorter than those in *L. seseloides* (1201 bp) and *P. japonicum* (1306 bp). Ls, *L. seseloides*; Pj, *P. japonicum*; Gl, *G. littoralis*.

Interspecies sequence variation was investigated by comparing multiple cp genome sequences of the three plant species. Most genic regions were well conserved compared with intergenic regions, except for several polymorphic genic regions found in *rpoC2*, *ycf1*, and *ycf2* (Figure S3). Three interesting, large structural changes specific to *P. japonicum* were identified, and one indel variation within an intergenic region was found in the cp genome of the three species (Figure 2 and Figure S3). A huge IR expansion was found in the *P. japonicum* cp genome (Figure 2A). Unlike *L. seseloides* and *G. littoralis*, the IR regions of *P. japonicum* were greatly expanded to a size of 35,759 bp–17,546 bp and 17,092 bp longer than those of *L. seseloides* (18,213 bp) and *G. littoralis* (18,667 bp), respectively. In *P. japonicum*, this IR expansion represents an increase in duplicated gene copies for 15 genes (*infA*, *petB*, *petD*, *rpl14*, *rpl16*, *rpl2*, *rpl22*, *rpl23*, *rpl36*, *rpoA*, *rps11*, *rps19*, *rps3*, *rps8*, and *ycf2*). We used PCR analysis to validate these expanded IR regions using the junction sequence between IR and LSC in *P. japonicum* cp (Figure 2C). Another structural change in *P. japonicum* compared to the other two species was a 625-bp fragment inversion, which resulted in three genes (*trnE*-UUC ~ *trnY*-GUA ~ *trnD*-GUC) being oppositely oriented (Figure 2D). Another indel was also identified near this inversion region on the *P. japonicum* cp genome, which was 454 bp and 435 bp shorter than that of *L. seseloides* and *G. littoralis*, respectively. For the *G. littoralis* cp genome, an indel variation was found at the intergenic target of *ycf2* ~ *trnL*-CAA within the IR region. The sequence length of this region in *G. littoralis* was 810 bp and 915 bp shorter than that of *L. seseloides* and *P. japonicum*, respectively (Figure 2E). Although these three species are relatively closely related, they show dynamic chloroplast genome rearrangement, especially in *P. japonicum*.

### 2.4. Tandem Repeats and Copy Number Variations in the Cp Genomes of Three Species 

By comparing the sequences of cp genomes at the interspecies level, 40 copy number variations (CNVs) of tandem repeat (TR) units were found (Table 2). In the cp genomes of *L. seseloides*, *P. japonicum*, and *G. littoralis*, 29, 22, and 11 TRs were found, respectively. TR units ranged in length from 10 to 39 bp, with 14-bp TR units being most abundant (7), followed by 17-bp units (4), and 18-bp units (4). Most of the 40 CNVs were present in intergenic regions, and only three were present in genic regions of the *ycf2* and *ycf15* genes. CNVs of various TR units related to indel polymorphism between the three species were also identified.

### 2.5. Sequence Variations of 45S DNA Sequences of Three Species 

Comparing the two accessions of each species analyzed in this study, their respective 45S nrDNA unit sequences were found to be identical (Table 1), except for a heterogeneous site (4,482 nucleotide position) with co-appearance of T and C in the *L. seseloides* 45S nrDNA unit sequence (Table S2). At the interspecies level, ITS1 and ITS2 sequences were highly polymorphic compared with the rRNA gene sequences. A total of 66 SNPs was found among the three plant species: 2 in 18S rRNA, 18 in ITS1, 1 in 5.8S rRNA, 12 in ITS2, and 33 in 26S rRNA regions (Table S2).

### 2.6. Phylogenetic Analysis and Divergence Time Estimation 

**Figure 3:**
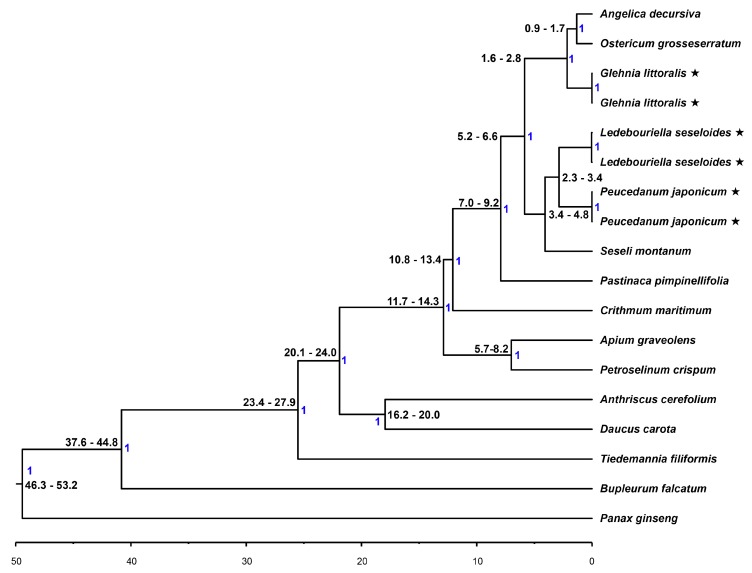
Phylogenetic relationship and estimated divergence time of 14 related Apiaceae species. A phylogenetic tree was constructed with BEAST, using 76 protein coding sequences common to cp genomes, including those of the *Panax ginseng* cp genome as an outgroup. Divergence times were estimated based on the divergence time (49 Mya) between *Daucus carata* (Apiaceae) and *P. ginseng* (Araliaceae). Posterior probability value indicated by blue color.

Using 76 protein-coding genes of 14 Apiaceae species, Bayesian phylogenetic inference analysis was conducted with BEAST (v. 2.4.3). Divergence time was calibrated based on the point at which the Apiaceae and Araliaceae families diverged 49.5 million years ago (Mya). *L. seseloides* and *P. japonicum* appeared to be in the same group, which diverged 2.3–3.4 Mya. However, *G. littoralis* was grouped with *Angelica decursiva* and *Ostericum grosseserratum* (Figure 3), which diverged between 1.6 and 2.8 Mya. It is estimated that these two groups branched out from common ancestors between 5.2 and 6.6 Mya. Phylogenetic analysis of 45S nrDNA also concurred with cp genome-based phylogeny (Figure S6).

### 2.7. Comparison of Mutation Rate among All Cp Protein-Coding Genes in the Three Apiaceae Species

**Figure 4:**
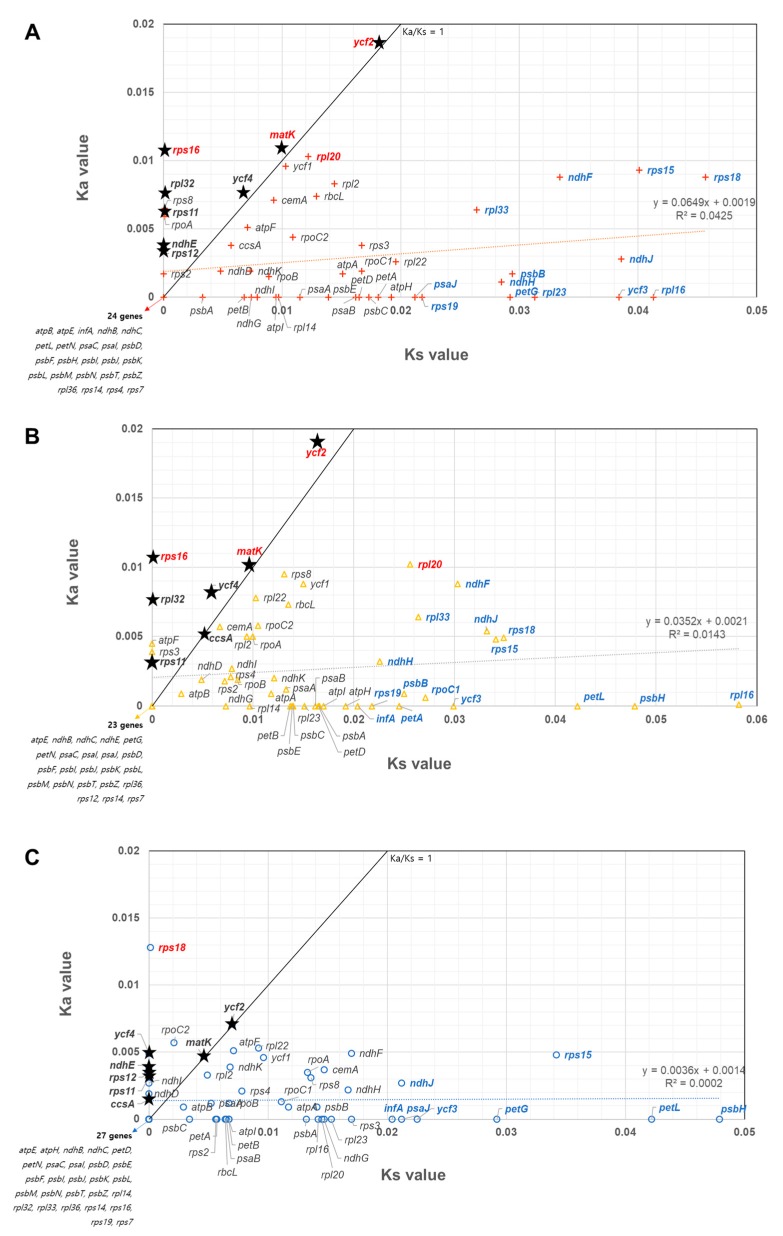
Chloroplast (cp) gene selection pressure of three Apiaceae species. Scatterplot of non-synonymous substitution (Ka) and synonymous substitution (Ks) values between *Ledebouriella seseloides* and *Glehnia littoralis* (**A**); between *Peucedanum japonicum and G. littoralis* (**B**); and between *L. seseloides* and *P. japonicum* (**C**). Ka and Ks values were calculated using CodeML and 76 protein-coding genes. Ka and Ks values are represented on the y-axis and x-axis, respectively. *R^2^* indicates a trend line. Red letters indicate genes with a Ka value greater than 0.01, and blue letters indicate genes with a Ks value greater than 0.02. Genes marked by an asterisk (*) are commonly positively selected genes in at least two analysis results. Ls, *L. seseloides*; Pj, *P. japonicum*; and Gl, *G. littoralis*.

To determine the mutation rate of the 76 protein-coding genes in the cp genome of the three tested plant species, we calculated non-synonymous substitution (Ka) and synonymous substitution (Ks) values for each gene and displayed them in a scatterplot. Supporting the phylogenetic analysis, the median values for Ka and Ks were 0.000 and 0.004 between *L. seseloides* and *P. japonicum*, respectively; 0.000 and 0.009 between *P. japonicum* and *G. littoralis*, respectively; 0.0000 and 0.0072 between *L. seseloides* and *G. littoralis* respectively (Figure 4). Genes with higher Ka than Ks values (Ka/Ks ≥ 1) represent positively selected genes during speciation. Between 9 and 11 genes were positively selected in each species. Among these, four genes, *matK*, *rpl20*, *rps16*, and *ycf2*, had high Ka values (over 0.01) between *G. littoralis* versus *P. japonicum/L. seseloides*, meaning that these genes might be actively involved in the divergence of *G. littoralis* from the others (Figure 4A,B). On the other hand, one gene, *rps18*, had a higher Ka value over 0.01 between *P. japonicum* and *L. seseloides* (Figure 4C). Sixteen genes showed higher neutral mutation (over 0.2 Ks values) between *G. littoralis* versus *P. japonicum*; 13 between *G. littoralis* versus *L. seseloides*; and eight between *P. japonicum* and *L. seseloides*. Meanwhile, 24, 23, and 27 genes were identical between *G. littoralis* versus *P. japonicum*, *G. littoralis* versus *L. seseloides*, and *P. japonicum* and *L. seseloides*, respectively. Nineteen genes were mutually identical among the three species: *ndhB*, *ndhC*, *petD*, *petN*, *psaC*, *psaI*, *psbD*, *psbF*, *psbI*, *psbJ*, *psbK*, *psbL*, *psbM*, *psbN*, *psbT*, *psbZ*, *rpl36*, *rps4*, and *rps14*. Five of these genes (*psbF*, *rpl36*, *petN*, *psbZ*, and *psbJ*) were well conserved among all 16 Apiaceae family species, while *psbF* was also conserved in *P. ginseng*, which belongs to the Araliaceae. Overall, nine genes, *ccsA*, *matK*, *ndhE*, *rpl32*, *rps11*, *rps12*, *rps16*, *ycf2*, and *ycf4*, provide more information for classification of the three species. 

### 2.8. Development of Barcode Markers Derived from cp Genomes and 45S nrDNA Sequences

Nine barcode markers were developed to discriminate between each of the three tested plant species (Table 3). These markers were based on length polymorphisms identified by comparing cp genome and 45S nrDNA unit sequences at the interspecies level. IR01 was designed from the 17-Kbp IR expansion observed in the *P. japonicum* cp genomes. The marker comprises three primers: one pair as controls and one LSC primer amplifying the newly expanded junction between LSC and IRs in *P. japonicum* (Figure 2B). The control primer amplifies all three plant species around 260 bp, while the reverse control primer site—located in the IRB and IRA sites of *P. japonicum*—reacts with the LSC primer and amplifies around 570 bp in *P. japonicum* only (because of the characteristic of this IR region) (Figure 2C). 

**Figure 5:**
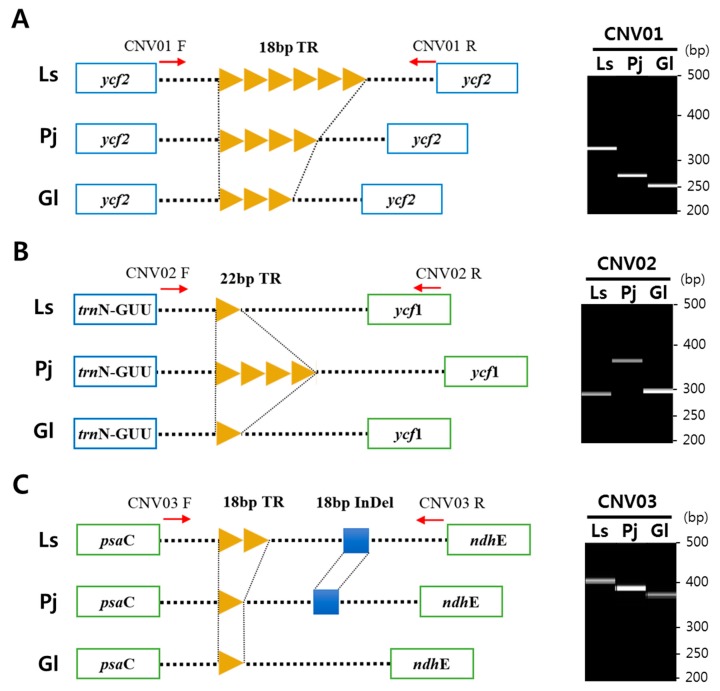
Schematic diagram and digital gel images from capillary electrophoresis of barcode markers derived from copy number variations (CNVs) of tandem repeat (TR) units in chloroplast genome. (**A**) Six, four, and three copies of an 18-bp TR unit were found in intergenic regions of *trnI-CAU ~ trnL-CAA* in *Ledebouriella seseloides*, *Peucedanum japonicum*, and *Glehnia littoralis*. (**B**) Four copies of a 22-bp TR unit were found in the intergenic region of *trnN-GUU ~ ycf1* in *P. japonicum*. (**C**) Two copies of an 18-bp TR unit were found in the intergenic region of *psaC ~ ndhE* in *L. seseloides*, and insertion of an 18-bp sequence was also found in the regions of *L. seseloides* and *P. japonicum*. Red arrows indicate primers used for PCR analysis. The size differences of PCR amplicons are shown on the right side of the schematic diagram. Ls, *L. seseloides*; Pj, *P. japonicum*; Gl, *G. littoralis*. CNV01, CNV02, and CNV03 indicate marker IDs.

Markers CNV01 and CNV03 can discriminate all three species with different sized amplicons (Figure 5A,C). CNV02, InDel01, InDel04, and IR01 amplified distinct amplicons in *P. japonicum*, by which the species could be clearly discriminated from other two species (Figure 2C and Figure S4). InDel02 and nrDNA01 amplified distinct amplicons in *G. littoralis*; thus, *G. littoralis* could be discriminated from the other two species (Figures S4 and S5). InDel03 generated a distinct amplicon only in *L. seseloides* to specifically detect this species. Although PCR amplicons with slightly different sizes (<3 bp) were identified among the three species, the size differences were ignored in this study because of the low separating power of electrophoresis using agarose gel. The marker nrDNA01 was designed to detect SNPs present only in ITS1 of *G. littoralis* 45S nrDNA sequences and was also successfully validated by PCR analysis (Figure S5). Proper combination of nine polymorphic markers can be applied for clear authentication of the three species. 

## 3. Discussion

### 3.1. Molecular Phylogeny of the Apiaceae Species

The taxonomic classification of Apiaceae species has not been established. The taxonomic classification system proposed by Drude [15] was based on broad inspection; for example, fruit morphology and anatomy. Though later modified, this approach remains widely accepted for discriminating Apiaceae species. Molecular classification based on DNA barcoding markers—an approach proposed by the Consortium for the Barcode of Life (CBOL)—proved to be an easy and accurate way of discriminating plant species and led to further clarifications of plant diversity and evolution. A previous study proposed that *Peucedanum* species are closely related to *Angelica* species [16]. Our data, which are based on complete cp genomes and 45S nrDNA, indicate that *G. littoralis* is actually more closely related to *Angelica* species.

### 3.2. Intraspecies Chloroplast Variation and DNA Barcoding Markers for Species Authentication 

Cp genomes are generally conserved and thus have been considered to have little polymorphism at the intraspecies level [9]. However, comparisons of diverse and complete cp genomes have revealed varying amounts of intraspecies polymorphism. The cp genomes of 14 ginseng accessions contained 12 polymorphisms: six indels and six SNPs [14]. Here, we identified intraspecies polymorphism in *L. seseloides* and *G. littoralis*, but not in *P. japonicum*. *L. seseloides* showed the most diverse intraspecies polymorphism: six SNPs and three InDels. *L. seseloides* is not indigenous to Korea, but rather was imported from China as a processed medicinal product. We assume that the two collections studied here originate from different locations in China. *G. littoralis* is indigenous to Korea and, between the two accessions collected from different eastern coastal areas of Korea, six polymorphisms were identified: five SNPs and one indel. By contrast, the two *P. japonicum* accessions analyzed had identical cp genome sequences, even though they were collected from different sites. Despite this evidence, we hesitate to conclude that *P. japonicum* has such narrow genetic diversity. Because this species is now cultivated in Korea and Japan, it is possible that the two individual plants sampled here may have been derived from the same cultivated genotype.

Intraspecies polymorphic sites are useful for the classification of a genotype within a species and can be utilized as specific markers for a cultivar, such as in the study of ginseng [14]. However, intraspecies polymorphic sites should be excluded for the development of barcoding markers because they may confuse the process of species authentication [17,18]. In this study, we developed nine barcoding markers that can authenticate each species by avoiding the intraspecies polymorphic sites for each species. We suggest using several markers together for species authentication to account for any as-yet unidentified intraspecies diversity. 

### 3.3. Unique Structural Changes in the cp Genome of P. japonicum 

Our data revealed dynamic structural changes in the cp genome of *P. japonicum*. Our cp genome sequence data showed that the *P. japonicum* and *L. seseloides* are more closely related, and *G. littoralis* is more diverged. However, *L. seseloides* and *G. littoralis* have common cp genome structures, while *P. japonicum* showed three unique structural changes as an expansion of IR sequences, inversion of three tRNA genes, and a deletion near to the inverted region. The inversion and deletion show no association but seemed to result from independent mutations (Figure 2A–C and Figure S3). 

Considering that IRs may be involved in rearrangement and stabilization of the cp genome [19], IR expansion might induce structural modifications in the *P. japonicum* cp genome, such as gene inversion in recent years (Figure 2D). Previously proposed hypotheses for possible mechanisms of IR expansion or contraction involve plastome rearrangement within cp genomes during evolution via recombination between poly A regions [20], repeated sequences [21], and/or duplication or deletion of a certain gene through inversion [22]. Such rearrangements may alter the stability of the genomic structure, which in turn could cause an IR boundary shift. The three structural changes described here for *P. japonicum* could have occurred together or could have been triggered by each other after divergence from *L. seseloides* 2.3–3.4 Mya (Figure 3).

### 3.4. Variation in the cp Genomes and 45S nrDNA of Three Species

Although the same genes were present in the cp genomes of the three tested plant species, they had many sequence variations at the interspecies level. As expected, most genic regions were more highly conserved than the intergenic regions in the cp genomes. However, some genic regions, such as *rpoC2*, *ycf1*, and *ycf2*, were polymorphic among the three species. Such genic regions have been used for barcoding regions in other plant species [13,14,23,24], indicating that these genic regions are hotspots of sequence polymorphism (Figure S3 and Table 2). Nineteen genes (*ndhB*, *ndhC*, *petD*, *petN*, *psaC*, *psaI*, *psbD*, *psbF*, *psbI*, *psbJ*, *psbK*, *psbL*, *psbM*, *psbN*, *psbT*, *psbZ*, *rpl36*, *rps4*, and *rps14*) were conserved among the three species; five of which (*psbF*, *rpl36*, *petN*, *psbZ*, and *psbJ*) were well conserved in the Apiaceae family, and one of them (*psbF*) remained in the Araliaceae. These genes were considered fundamental genes in Apiales cp genome evolution, and thus not suitable for classifying species.

The 45S nrDNA sequences generally show high variation in their ITS1 and ITS2 regions [25]. In the current study, rich variations were observed in 26S rDNA, followed by ITS1, ITS2, 18S rDNA, and 5.8S rDNA. Nevertheless, ITS1 and ITS2 regions remained efficient regions in which to search for DNA barcoding markers because they are simple and quick for PCR validation using universal primer sets. However, our data suggest that the 26S rDNA gene region may also be a good candidate to target for barcoding regions [26].

### 3.5. Cp Gene Selection Pressure and Phylogenetic Relationship of Three Apiaceae Species

The ratio between nonsynonymous (Ka) and synonymous (Ks) nucleotide substitution has been widely used in studies to compare genome or gene evolution rates [27]. Overall, Ka and Ks values were higher between *G. littoralis* and either *P. japonicum* or *L. seseloides* than between *P. japonicum* and *L. seseloides*. The genes *rps16*, *matK*, and *ycf2* were under positive selection, showing the highest Ka values, over 0.01, between *G. littoralis* and the other two species. We conclude that these three genes might have been actively involved in the divergence of *G. littoralis* from the other species (Figure 4A,B). However, only one gene, *rps18*, showed positive selection, with a Ka value over 0.01, between *P. japonicum* and *L. seseloides* (Figure 4C). These positively selected genes may be used more effectively for species classification in Apiaceae species (Figure 4). In fact, *ycf2* was used to distinguish between these three species (Figure 2E). 

Many studies have reported phylogenetic relationships between members of the Apiaceae family using partial cp genic regions such as *rpoC1*, *rpl16*, *matK*, and *rbcL* [16,28,29,30], intergenic regions of *trnH*-*psbA*, and *trnQ*-*trnK* [31,32], and ITS regions of 45S nrDNA [33,34,35]. Nevertheless, to date, there has been no comprehensive phylogenic study of these three Apiaceae species, *L. seseloides*, *P. japonicum* and *G. littoralis*. Here, we used complete cp and 45S nrDNA sequence information to phylogenetically analyze the three species and other Apiaceae. Although *L. seseloides* and *G. littoralis* were more structurally similar, and *P. japonicum* showed three unique structural variations, our phylogenetic analysis revealed that *L. seseloides* was more closely related to *P. japonicum* than to *G. littoralis* (Figure 4). 

Molecular clock analysis will be interesting to see how the unique cp genome structures changes in the *P. japonicum* cp genome during this period. Inspection of more cp genomes of other species that are closely related to *P. japonicum* should clarify to reveal how and when the chloroplast genome structure was changed in *P. japonicum* lineage.

## 4. Materials and Methods 

### 4.1. Plant Materials

In this study, we conducted sequencing and assembly of the cp genomes and 45S nrDNA of three Apiaceae species, *L. seseloides*, *P. japonicum*, and *G. littoralis*. Two individual plant samples of each species were collected; one from each of two different locations (Table 1). *L. seseloides* plants were collected from China, and *P. japonicum* and *G. littoralis* were collected from South Korea. All plants were maintained in the medicinal plant gardens at Seoul National University and the Ministry of Food and Drug Safety, South Korea. 

### 4.2. Genomic DNA Preparation and Whole-Genome Shotgun Sequencing

Total genomic DNA was extracted from leaves using a modified cetyltrimethylammonium bromide (CTAB) method [36]. The quantity and quality of genomic DNA was examined using a UV-spectrophotometer (Thermo Scientific Nanodrop ND-1000, Waltham, MA, USA) and agarose gel electrophoresis. For WGS, genomic libraries with a 300-bp insert size were prepared according to the standard paired-end (PE) protocol (Illumina, San Diego, CA, USA) and sequenced by the National Instrumentation Center for Environmental Management (http://nature.snu.ac.kr/kr.php), Seoul, Korea, using an Illumina genome analyzer (HiSeq2000). The library for each plant sample was separately tagged with a different Illumina index and pooled for sequencing in a single lane. After sequencing 101 cycles, PE reads for each plant sample were collected according to the index.

### 4.3. Cp Genome and 45S nrDNA Assembly

We assembled complete cp genome sequences of accessions Ls-01 (KT153021) and Gl-01 (KT153022) in previous work [37,38]. Complete cp genomes and 45S nrDNA sequences of the remaining samples were generated using the dnaLCW method, as described by Kim et al. [13,14]. In brief, trimmed, high-quality reads with Phred scores of 20 or greater were obtained from the total PE reads using the CLC-quality trim tool and then were assembled using a CLC genome assembler (version 4.06 beta, CLC Inc, Aarhus, Denmark). 

Initial contigs of the cp genome were selected using *Panax ginseng* (KM088019) [14] and *Daucus carota* (NC_008325) [39] as reference sequences with MUMmer [40]. cp contigs were then ordered based on reference cp sequences and merged into a single draft sequence by connecting overlapping terminal sequences. The draft cp sequences were curated manually by re-mapping raw WGS reads. Average coverage of mapped reads ranged between 146.53x and 945.25x (Table 1 and Figure S1).

For 45S nrDNA assembly, the longest initial contigs, including the 45S cistron unit, were selected by comparing with reported 45S nrDNA sequences of *Panax ginseng* (KM036295). The average coverage of raw PE reads mapped to 45S nrDNA sequences ranged between 399.28× and 2550.62× (Table 1 and Figure S2). Assembly errors and gaps found in the draft sequences were manually corrected by mapping raw WGS reads.

### 4.4. Gene and Structural Annotation

The cp genes were annotated using DOGMA (http://dogma.ccbb.utexas.edu/) [41], with manual curation based on BLASTN searches. Circular maps of each cp genome were drawn using OGDRAW (http://ogdraw.mpimp-golm.mpg.de/) [42]. The structures of 45S nrDNA sequences were predicted by RNAmmer (http://www.cbs.dtu.dk/services/RNAmmer/) and BLASTN searches against the National Center for Biotechnology Information nucleotide database (https://blast.ncbi.nlm.nih.gov/Blast.cgi).

### 4.5. Comparative Sequence Analysis at the Intraspecies and Interspecies Level

To characterize intraspecies and interspecies variation, six cp genome and 45S nrDNA sequences were aligned with MAFFT (http://mafft.cbrc.jp/alignment/server/) [43], and sequence variations were visualized using mVISTA (http://genome.lbl.gov/vista/mvista/submit.shtml) [44] with the Shuffle LAGAN alignment program [45] and the BlastZ tool [46]. Some misaligned regions were manually curated using BioEdit (http://www.mbio.ncsu.edu/bioedit/bioedit.html). Then, polymorphic regions showing SNPs and indels were investigated at the intraspecies and interspecies levels. 

### 4.6. Development and Validation of DNA Barcode Markers

Using Primer 3 (http://bioinfo.ut.ee/primer3-0.4.0/), primers were designed from polymorphic sites of those cp genomes and 45S nrDNA sequences with interspecies diversity but no intraspecies variation. Amplification of marker was implemented with 20 ng of genomic DNA from three species in a 25 µL reaction volume containing 10× buffer, dNTP and Taq mixture (Inclone biotech, Gyonggido, Korea) with 10 pmol of each primer. Amplification was performed on Veriti 96-Well Thermal Cycler (Advanced Analytical Technologies Inc., Santa Clara, CA, USA) under the following conditions: initial denaturation at 94 °C for 5 min, followed by 35 cycles of denaturation at 94 °C for 90 s, annealing at 54 °C for 90 s, and extension at 72 °C for 90 s, and final extension at 72 °C for 7 min. Amplified PCR fragments were analyzed with electrophoresis by 1.3% agarose gels at 100 V for 20 min, and also with a capillary electrophoresis instruments known as fragment analyzer, with DNF- 905 Kit (Advanced Analytical Technologies Inc.), under following condition according to the manufacturer’s instructions. Digital gel images of amplificon were generated from Prosize 2.0 program (Advanced Analytical Technologies Inc. https://www.aati-us.com/support/software/).

### 4.7. Calculation of Nucleotide Substitution Value

The non-synonymous substitution (Ka) to synonymous substitution (Ks) ratio was calculated with the maximum likelihood method using CodeML (Berkeley, CA, USA, version 4.9 h) [47]. To avoid duplication, only one copy of the gene in IRs was used in the analysis. For *ycf1*, the difference in length of smaller copies was eliminated. The average values of Ka, Ks, and Ka per Ks were calculated for 76 protein-coding genes (Supplementary Data S1).

### 4.8. Phylogenetic Analysis

Phylogenetic relationships between cp genomes were estimated using 15 species (representing 18 accessions) in the Apiales order (Apiaceae + Araliaceae) as follows: *Angelica decursiva* (KT781591), *Anthriscus cerefolium* (NC_015113), *Apium graveolens* (NC_041087), *Bupleurum falcatum* (NC_027834), *Crithmum maritimum* (HM596072), *D. carota* (NC_008325), *G. littoralis* (KT153022, KU866532), *L. seseloides* (KT153021, KU866529), *O. grosseserratum* (KT852844), *P. ginseng* (NC_006290), *Pastinaca pimpinellifolia* (NC_027450), *Petroselinum crispum* (HM596073), *P. japonicum* (KU866530, KU866531), *Seseli montanum* (NC_027451), and *Tiedemannia filiformis* (HM596071). Phylogenetic analysis was conducted in Yule speciation prior, GTR + I + Γ substitution, and a strict clock model using BEAST (Auckland, New Zealand, version 2.4.3) [48]. Two independent Markov chain Monte Carlo runs were also performed for 10,000,000 generations, and trees were sampled every 100 steps. We used the same 76 protein-coding genes as in the above nucleotide substitution analysis. Phylogeny of 45S nrDNA was constructed to support relationships between the three Apiaceae species (*L. seseloides*, *P. japonicum*, and *G. littoralis*) using the same programs and parameters as mentioned above.

## Figures and Tables

**Table 1:** Summary of whole-genome sequences generated and assembly status for three Apiaceae species.

Species (Common Name in Traditional Korean Medicine)	Accession	Raw Data (Gbp)	Chloroplast Genome			45S nrDNA		
			Length (bp)	Coverage (x) ^d^	GenBank Accession No. (Reference)	Length (bp) ^e^	Coverage (x) ^d^	GenBank Accession No. (Reference)
*Ledebouriella seseloides* (Bang-poong)	Ls-01 ^a^	1.67	147,880	255.15	KT153021 (Lee et al., 2015a)	5815	659.94	KX757774 (This study)
	Ls-02 ^b^	5.05	147,830	177.95	KU866529 (This study)	5815	2550.62	KX757775 (This study)
*Peucedanum japonicum* (Sik-bang-poong)	Pj-01 ^a^	2.08	164,653	369.41	KU866530 (This study)	5815	943.43	KX757776 (This study)
	Pj-02 ^b^	5.18	164,653	945.25	KU866531 (This study)	5815	2418.35	KX757777 (This study)
*Glehnia littoralis* (Hae-bang-poong)	Gl-01 ^c^	1.06	147,467	146.53	KT153022 (Lee et al., 2015b)	5812	399.28	KX757778 (This study)
	Gl-02 ^b^	4.72	147,477	367.41	KU866532 (This study)	5812	1638.57	KX757779 (This study)

Leaf samples were provided by ^a^ Department of Herbal Crop Research, National Institute of Horticultural and Herbal Science, Rural Development Administration, Korea (http://www.nihhs.go.kr/); ^b^ Korea Food & Drug Administration, Cheongju-si, Chungcheongbuk-do, Korea; and ^c^ Yeongdeok-gun Country Office, Yeongdeok-gun, Gyeongsangbuk-do, Korea. ^d^ Coverage of chloroplast (cp) genome and 45S nrDNA indicates the total WGS read depth used to assemble the complete sequences. ^e^ Length of the 45S nrDNA unit sequence with a complete cistron unit.

**Table 2:** Copy number variations of tandem repeat (TR) units in chloroplast (cp) genomes among three Apiaceae species.

No.	TR Unit Sequence	Unit Length (bp)	Copy Number			Position
			Ls	Pj	Gl	
1	AATAAGTAACTAG	13	2	1	1	*trn*K-UUU ~ *rps*16
2	TTCTTCTATACAAA	14	2	2	2	*rps*16 ~ *trn*Q-UUG
3	AAAGATATGATTCATA	16	2	2	1	*rps*16 ~ *trn*Q-UUG
4	TAAAAAATATAAA	13	2	1	1	*atp*F ~ *atp*H
5	AGGAATCATTAAA	13	2	1	1	*rps*2 ~ *rpo*C2
6	TATATTGTTATAAT	14	1	1	2	*rpo*B ~ *trn*C-GCA
7	TATAATATTAATAAG	15	2	nf	nf	*trn*E-UUC ~ *trn*T-GGU
8	TATATAGGAAGTATGAT	17	nf	2	1	*trn*S-UGA ~ *lhb*A
9	ATCTAGATAAGATTTATA	18	2	1	1	*trn*M-CAU ~ *psb*D
10	TTAAATGGTATTTATTAAT	19	2	1	1	*trn*S-UGA ~ *lhb*A
11	ATTAGCCATTACTAATAG	18	1	2	1	*psa*A ~ *ycf*3
12	TAACCTAAGTGCAAAAATAGA	21	1	2	1	*ycf*3 ~ *trn*S-GGA
13	TATTCTATATATATATATTCTATATA	26	1	2	1	*rps*4 ~ *trn*T-UGU
14	TTTATATATATATATAT	17	2	1	1	*ndh*C ~ *trn*V-UAC
15	TTATCTTTATAATTTAA	18	1	2	1	*acc*D ~ *psa*I
16	TATATGTATATTGA	14	2	2	1	*psa*I ~ *ycf*4
17	TTATTCCAGTAAAA	14	2	2	2	*cem*A ~ *pet*A
18	AAGGAAGTACTC	12	1	2	1	*psb*E ~ *pet*L
19	ATTATATATAT	11	nf	3	1	*trn*W-CCA ~ *trn*P-UGG
20	TTATACAAGGTACTTAAATGTAAA	24	2	1	1	*psa*J ~ *rpl*33
21	TTCTATATAGAACATAATTAAATA	24	2	2	2	*rpl*33 ~ *rps*18
22	TAAAGTTCCAACTAAAAAG	19	2	1	2	*psb*T ~ *psb*N
23	TCCCCTTTTT	10	1	2	1	*rps*11 ~ *rpl*36
24	ATATTTTTAAATTG	14	2	2	3	*rpl*16 ~ *rps*3
25	TCTTATAGAATTAGAATTGT	20	2	1	1	*rpl*16 ~ *rps*3
26	CCTATTGCCGATACA	15	2	2	2	*ycf*2
27	TAGTGACGATATTGATGC	18	6/5 ^a^	4	3	*ycf*2 (marker ID CNV01)
28	AAACTCTCTTCAAGAGTTATTAACACCAACCCGGTGTTC	39	nf	2	nf	*ycf*2 ~ *trn*L-CAA
29	TGGTGGAGATCAGAAAGAGAA	21	2	1	nf	*ycf*2 ~ *trn*L-CAA
30	TGTAATGTACTT	12	2	1	1	*ycf*2 ~ *trn*L-CAA
31	TCTTTTCTTCCGTGATGAACT	21	3	1	1	*ycf*15
32	ACCGAAAGGAAAAGCGTGAA	20	2	2	3	*trn*I-GAU ~ *trn*I-GAU
33	CATTGTTCAACTCTTTGACAACACGAAAAAAC	32	2	2	2	*rrn*4.5 ~ *rrn*5
34	AAAAAGAAATAAAT	14	2	2	1	*trn*R-ACG
35	TTCTATTTCTTTTCTATATATG	22	1	4	1	*trn*N-GUU ~ *ycf*1 (marker ID CNV02)
36	TATTAATATTAATAAAT	17	2	nf	nf	*ycf*1 ~ *ndh*F
37	TATATATGTATAAA	14	2	nf	nf	*rpl*32 ~ *trn*L-UAG
38	AAATATTAATCTACTTCT	18	2	1	1	*psa*C ~ *ndh*E (marker ID CNV03)
39	TTATGAATATAA	12	2	2	2	*psa*C ~ *ndh*E
40	TATTATTTTTTATTA	15	2	1	1	*ndh*A ~ *orf*188

^a^ Copy number variations at the intraspecies level. Ls, *Ledebouriella seseloides*; Pj, *Peucedanum japonicum*; and Gl, *Glehnia littoralis*. nf, not found.

**Table 3:** Barcode markers developed in this study.

Marker ID	Primer Sequence (5′–3′)	Product Size ^a^	Location
		Ls/Pj/Gl (bp)	
CNV01	F: GGTCAAATACCTAGCGACAA	308/272/254	*ycf2*
	R: TTATGCAAGGAGACATTGCT		
CNV02	F: CATCCATATCCCAATTCCAT	305/371/305	*trnN-GUU ~ ycf1*
	R: GCTCGGAGAAGGAAGAGATA		
CNV03	F: CATTGAGTGCACCCTATACA	404/386/368	*psaC ~ ndhE*
	R: TCGTAACAGAAAATCAACTCG		
InDel01	F: AACGAATCCTACGGTTTCTC	266/289/269	*trnS-GCU ~ trnR-UCU*
	R: TGTCGAACAGGGATAATTTG		
InDel02	F: TCTCGCTTTTTAGTCAGTTTG	380/379/416	*ndhF ~ rpl32*
	R: GCCTAATGAAAAGCCTAATGA		
InDel03	F: CAGGAGGATAGCAAGTTACAA	412/571/571	*rps12 ~ trnV-GAC*
	R: CAACGCCACTATTCTTGAAC		
InDel04	F: CTATATGTATATACAATAACGAATCA	652/198/633	*trnE-UUC ~ trnT-GGU*
	R: GTTCAAGAATAGTGGCGTTG		
IR01	LSC P: CCTAGCTGCTGTTGAAGCTC	na/576/na	psbA
	Control F: GACGACTGAGCCAACTTGAT	262/262/262	*psbH ~ petB*
	Control R: TCGAGACGTTCTTCAAACCA		
nrDNA01	Specific F: GTTAACAATTAGGGCGAGCA	na/na/291	ITS1
	Control F: GCATCGATGAAGAACGTAGC	78/78/78	5.8S
	Control R: GCGTTCAAAGACTCGATGGT		

^a^ Amplicon size expected for each of the three species. Ls, *Ledebouriella seseloides*; Pj, *Peucedanum japonicum*; and Gl, *Glehnia littoralis*. na, not amplified.

## Supplementary Materials

Supplementary materials can be found at https://www.mdpi.com/1422-0067/20/9/2196/s1. Figure S1. Complete cp genome assemblies of *L. seseloides* (**A**), *P. japonicum* (**B**), and *G. littoralis* (**C**). Figure S2. Schematic diagram of 45S nrDNA cistron unit of *L. seseloides* (**A**), *P. japonicum* (**B**), and *G. littoralis* (**C**). Figure S3. Comparison of cp genome sequences of three Apiaceae species at inter-species level. Figure S4. Validation of InDel markers. Figure S5. Development and validation of barcode marker nrDNA01 derived from *G. littoralis* 45S nrDNA unit sequence. Figure S6. Phylogenetic tree of three Apiaceae species using 45S nrDNA sequence. Table S1. Gene contents of cp genomes in three Apiaceae species. Table S2. Cp genome sequence variation of *L. seseloides* between two accessions at the intraspecies level. Table S3. Cp genome sequence variation of *G. littoralis* between two accessions at the intraspecies level. Supplementary Data S1: Ka and Ks analysis results between the three species.
