# Supplementary material for: Dynamic Chloroplast Genome Rearrangement and DNA Barcoding for Three Apiaceae Species Known as the Medicinal Herb “Bang-Poong”
Source: Int J Mol Sci. 2019 May 4;20(9):2196. doi: 10.3390/ijms20092196 (PMC6539805; doi:10.3390/ijms20092196)
Supplement: Supplementary file 1 [file ijms-20-02196-s001.zip › ijms-469380-supplementary.docx]

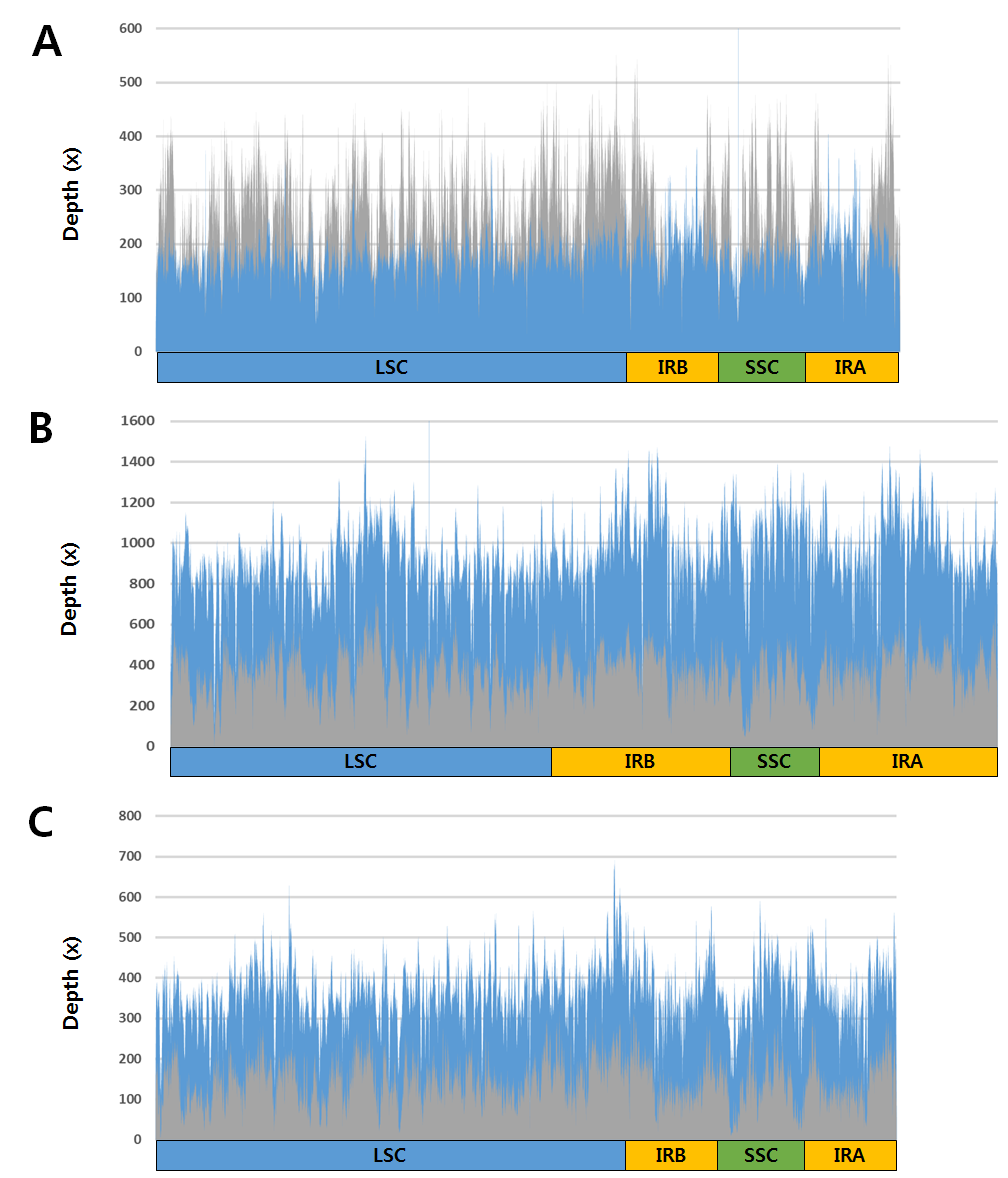


**Figure S1.** Complete cp genome assemblies of *L. seseloides* (A), *P. japonicum* (B), and *G. littoralis* (C). Mapping of raw PE reads on the assembled complete cp genome. The overall structure of the cp genome is shown with different colored bars: blue, yellow and green for LSC, IRs, and SSC, respectively. Gray and blue lines indicate the mapping depth of accession nos. 01 and 02, respectively.


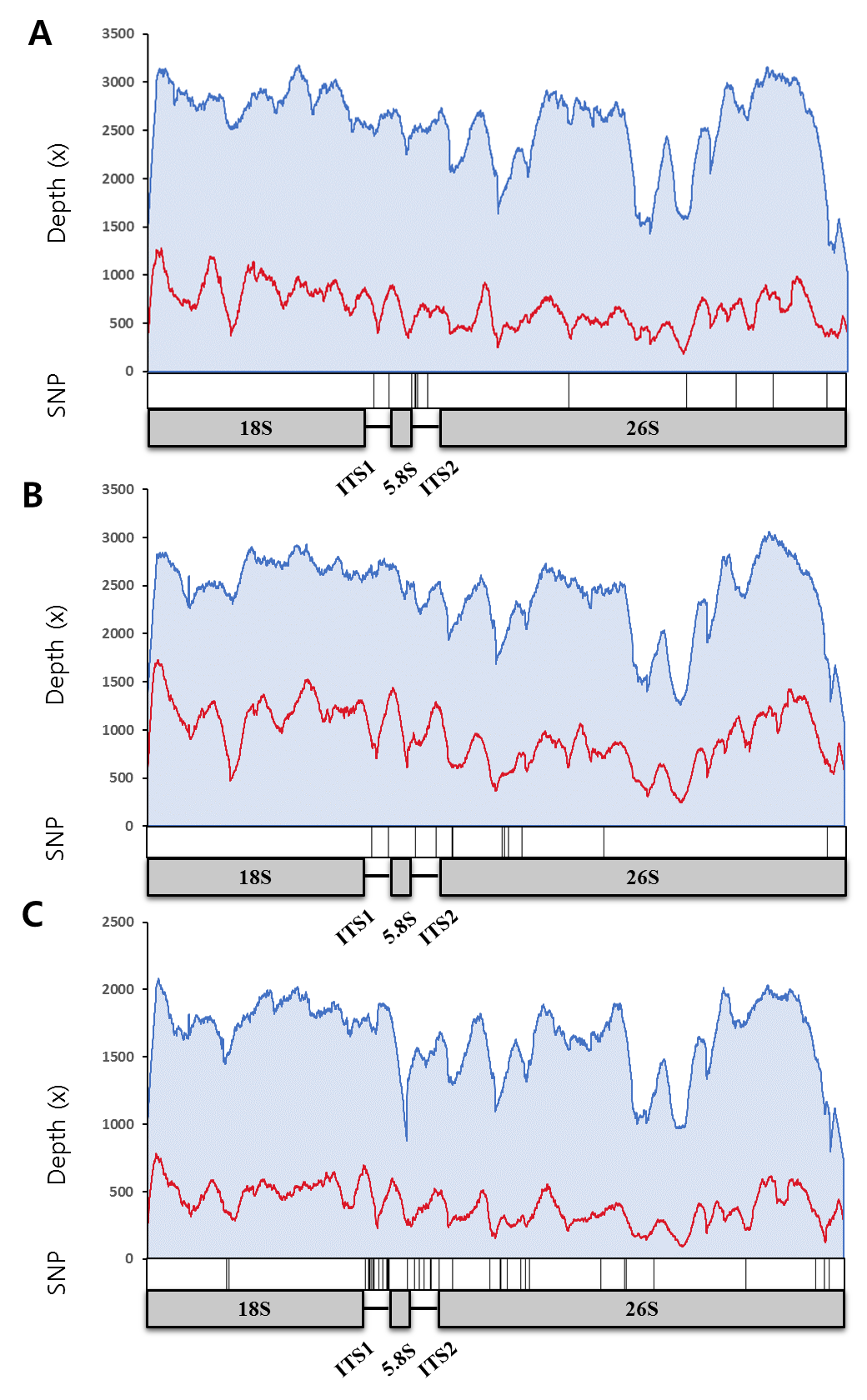


**Figure S2.** Schematic diagram of 45S nrDNA cistron unit of *L. seseloides* (A), *P. japonicum* (B), and *G. littoralis* (C). Mapping of raw PE reads on the assembled 45S nrDNA cistron sequence. Red and blue lines indicate the mapping depth of accession nos. 01 and 02, respectively. Black lines indicate the inter species SNP positions found among the three Apiaceae species.

**Figure S3.** Comparison of cp genome sequences of three Apiaceae species at inter-species level. Comparative map was generated using mVISTA with cp genome of *P. japonicum* (accession no Pj-01) as a reference. Three major polymorphic sites (**A**–**C**) are indicated. (**A**) Gene inverted site found only in *trnD-GUC ~ trnY-GUA ~ trnE-UUC* of P. japonicum. (**B**) Short intergenic region of *ycf2 ~ trnL-CAA* found only in G. littoralis. (**C**) Expanded IR regions found only in *P. japonicum*. Accession no of each species are indicated on the left of map. Blue bar, protein-coding region; red bar, intron; sky-blue bar, tRNA or rRNA.


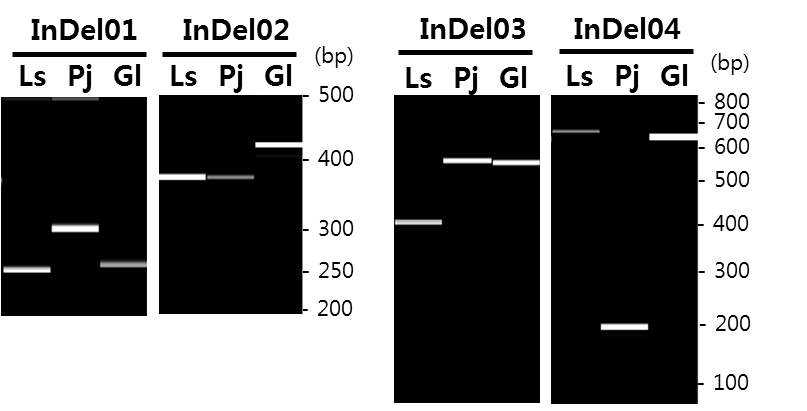


**Figure S4.** Validation of InDel markers. Four InDel markers, InDel01, InDel02, InDel03, and InDel04, were designed based on cp genome sequences of three Apiaceae species and validated successfully by PCR amplification. Digital gel images were generated by capillary electrophoresis. Ls, *L. seseloides*; Pj, *P. japonicum*; Gl, *G. littoralis*.


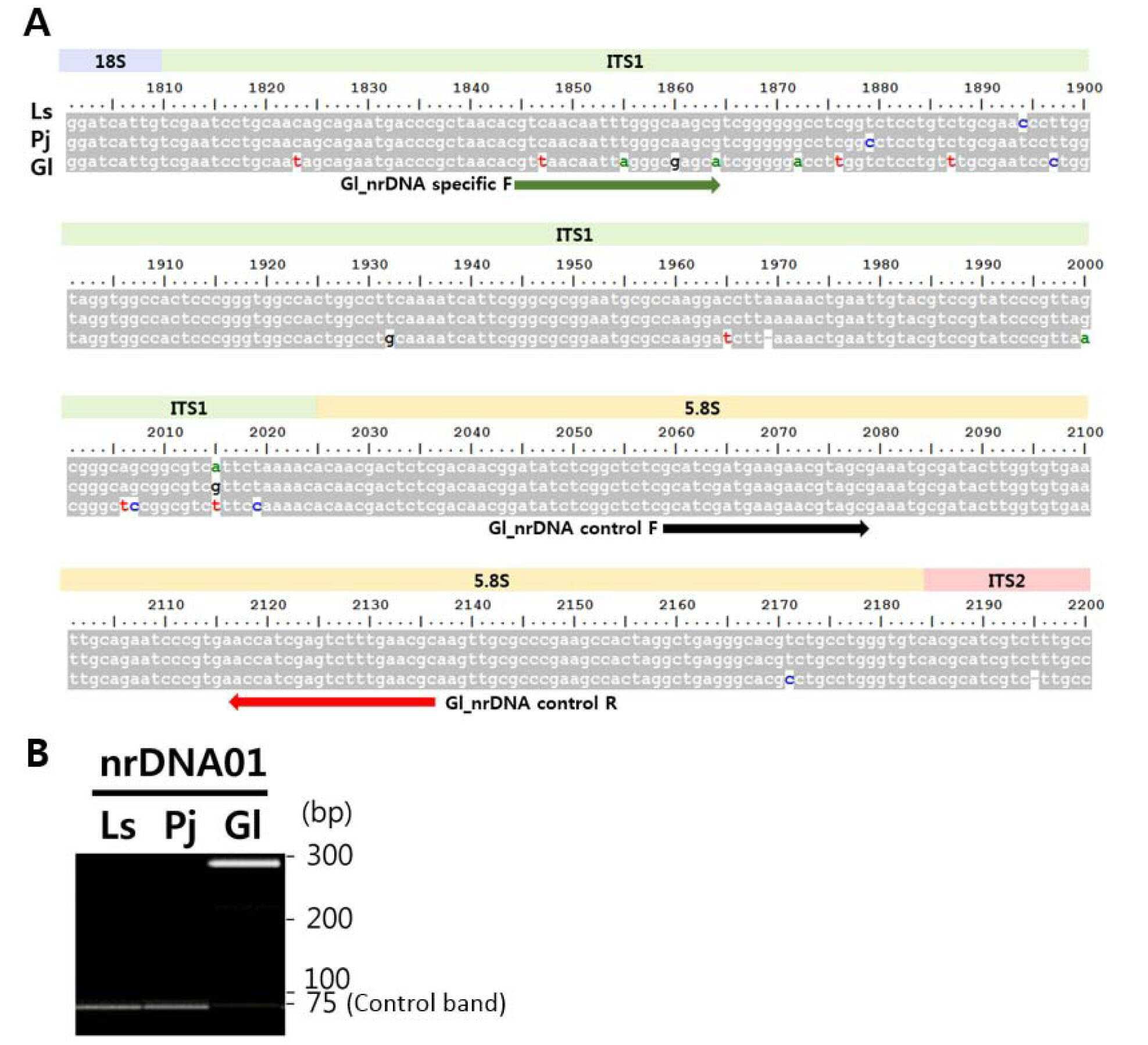


**Figure S5.** Development and validation of barcode marker nrDNA01 derived from *G. littoralis* 45S nrDNA unit sequence. (A) Schematic diagram of primer location. Green arrow indicates specific primer only to ITS1 of *G. littoralis* 45S nrDNA unit sequences, while black and red arrow indicate control primers designed based on 5.8S rRNA sequences common to all three Apiaceae species. (B) PCR validation of nrDNA01 marker. DNA fragment of about 300 bp was specifically amplified only in *G. littoralis*, while DNA fragment of about 75 bp amplified by control primers was present in all three Apiaceae species. Digital gel image were generated by capillary electrophoresis. Ls, *L. seseloides*; Pj, *P. japonicum*; Gl, *G. littoralis*.


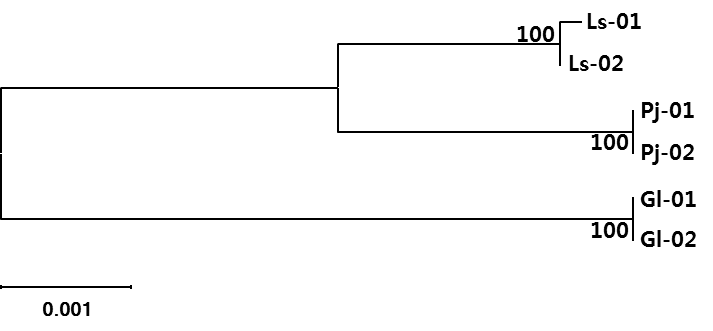


**Figure S6.** Phylogenetic tree of three Apiaceae species using 45S nrDNA sequence. Maximum Likelihood (ML) phylogeny were generated with 1,000 replications of bootstrap by MEGA6 program. Ls, *L. seseloides*; Pj, *P. japonicum*; Gl, *G. littoralis*.

**Table S1.** Gene contents of cp genomes in three Apiaceae species.

| **Category** | **Gene group** | **Gene name** |
| --- | --- | --- |
| Self-replication | Transfer RNAs | *trnA-UGC^1)3)^, trnC-GCA, trnD-GUC, trnE-UUC, trnF-GAA,*  *trnG-UCC^1)^, trnG-GCC, trnH-GUG,* ***trnI-CAU^5)^****, trnI-GAU^1)3)^,*  *trnK-UUU^1)^, trnL-UAA, trnL-UAG, trnL-CAA^3)^, trnM-CAU,*  *trnfM-CAU, trnN-GUU^3)^, trnP-UGG, trnQ-UUG, trnR-ACG^3)^,*  *trnR-UCU, trnS-GCU, trnS-GGA, trnS-UGA, trnT-GGU,*  *trnT-UGU, trnV-UAC^1)^, trnV-GAC^3)^, trnW-CCA, trnY-GUA* |
|  | Ribosomal RNAs | *rrn16^3)^, rrn23^3)^, rrn4.5^3)^, rrn5^3)^* |
|  | Ribosomal proteins small subunit | *rps2,* ***rps3^5^****^)^, rps4, rps7^3)^,* ***rps8^5^****^)^,* ***rps11^5^****^)^, rps12^2) 3) 4)^, rps14,*  *rps15, rps16, rps18,* ***rps19^5^****^)^* |
|  | Ribosomal proteins large subunit | ***rpl2^1^****^)^* ***^5^****^)^,* ***rpl14^5^****^)^,* ***rpl16^1^****^)^* ***^5^****^)^, rpl20,* ***rpl22^5^****^)^****, rpl23^5^****^)^, rpl33,* ***rpl36^5^****^)^* |
|  | RNA polymerase | ***rpoA^5^****^)^, rpoB, rpoC1, rpoC2* |
| Photosynthesis | Translational initiation factor | ***infA^5^****^)^* |
|  | Photosystem I | *psaA, psaB, psaC, psaI, psaJ, ycf3^2)^, ycf4* |
|  | Photosystem II | *psbA, psbB, psbC, psbD, psbE, psbF, psbH, psbI, psbJ, psbK, psbL, psbM, psbN, psbT, psbZ* |
|  | Cytochrome b6/f complex | *petA,* ***petB^6^****^)^,* ***petD^5^****^)^, petG, petL, petN* |
|  | ATP synthase | *atpA, atpB, atpE, atpF^1)^, atpH, atpI* |
|  | Large subunit of Rubisco | *rbcL* |
|  | NADH dehydrogenase | *ndhA^1)^, ndhB^1) 3)^, ndhC, ndhD, ndhE, ndhF, ndhG, ndhH, ndhI, ndhJ, ndhK* |
| Other genes | Maturase | *matK* |
|  | Envelope membrane protein | *cemA* |
|  | Subunit of acetyl-CoA | *accD* |
|  | C-type cytochrome synthesis gene | *ccsA* |
|  | Protease | *clpP* |
|  | Unknown function protein coding gene | *ycf1^3)^,* ***ycf2^5^****^)^, ycf15^3)^* |

^(1)^ Gene containing a single intron

^(2)^ Gene containing two introns

^(3)^ Two copies of gene in IRs

^(4)^ *Trans*-splicing gene

^(5)^ Two copies of gene in *P. japonicum*

^(6)^ Two copies of exon no. 2 in *P. japonicum*

**Table S2.** Summary of single nucleotide polymorphisms (SNPs) found in 45S nrDNA sequences among the three Apiaceae species.

| 45S nrDNA subunits | 18S | | ITS1 | | | | | | | | | | | | | | | | | | 5.8S | | ITS2 | | | | | | | | | | | | | | | | | | | | | |
| --- | --- | --- | --- | --- | --- | --- | --- | --- | --- | --- | --- | --- | --- | --- | --- | --- | --- | --- | --- | --- | --- | --- | --- | --- | --- | --- | --- | --- | --- | --- | --- | --- | --- | --- | --- | --- | --- | --- | --- | --- | --- | --- | --- | --- |
| **Nucleotide position^a^** |  |  | 1 | 1 | 1 | 1 | 1 | 1 | 1 | 1 | 1 | 1 | 1 | 1 | 1 | 2 | 2 | 2 | 2 | 2 | 2 | | 2 | 2 | 2 | 2 | | 2 | | 2 | | 2 | | 2 | | 2 | | 2 | | 2 | | 2 | |  |
|  | 6 | 6 | 8 | 8 | 8 | 8 | 8 | 8 | 8 | 8 | 8 | 8 | 8 | 9 | 9 | 0 | 0 | 0 | 0 | 0 | 1 | | 2 | 2 | 2 | 2 | | 2 | | 2 | | 3 | | 3 | | 3 | | 3 | | 3 | | 4 | |  |
|  | 6 | 8 | 2 | 4 | 5 | 6 | 6 | 7 | 7 | 7 | 8 | 9 | 9 | 3 | 6 | 0 | 0 | 0 | 1 | 1 | 7 | | 0 | 3 | 3 | 4 | | 5 | | 6 | | 1 | | 3 | | 6 | | 6 | | 7 | | 1 | |  |
|  | 8 | 5 | 3 | 7 | 5 | 0 | 4 | 2 | 6 | 9 | 7 | 4 | 7 | 2 | 5 | 0 | 6 | 7 | 5 | 9 | 1 | | 5 | 0 | 1 | 1 | | 0 | | 8 | | 1 | | 8 | | 4 | | 8 | | 2 | | 2 | |  |
| ***Ledebouriella seseloides*** | T | G | C | C | T | A | G | G | C | T | C | **C** | T | T | C | G | A | G | **A** | T | T | | **G** | T | **A** | **G** | | **A** | | G | | A | | **C** | | T | | A | | A | | C | |  |
| ***Peucedanum japonicum*** | T | G | C | C | T | A | G | G | C | **C** | C | T | T | T | C | G | A | G | **G** | T | T | | A | T | C | **A** | | G | | G | | A | | G | | T | | A | | A | | **T** | |  |
| ***Glehnia littoralis*** | **A** | **T** | **T** | **T** | **A** | **G** | **A** | **A** | **T** | T | **T** | T | **C** | **G** | **T** | **A** | **T** | **C** | **T** | **C** | **C** | | A | **C** | C | **-** | | G | | **T** | | **G** | | G | | **C** | | **C** | | **C** | | C | |  |
|  | | | | | | | | | | | | | | | | | | | | | | | | | | | | | | | | | | | | | | | | | | | | |
| **45S nrDNA subunits** | **26S** | | | | | | | | | | | | | | | | | | | | | | | | | | | | | | | | | | | | | | | | | | | |
| **Nucleotide position ^a^** | 2 | 2 | 2 | 2 | 2 | 2 | 2 | 2 | 2 | 2 | 3 | 3 | 3 | 3 | 3 | 3 | 3 | 3 | 3 | 3 | 3 | 3 | 4 | 4 | | | 4 | | 4 | | 5 | | 5 | | 5 | | 5 | | 5 | | 5 | | 5 | |
|  | 4 | 5 | 5 | 5 | 8 | 9 | 9 | 9 | 9 | 9 | 0 | 0 | 1 | 1 | 1 | 1 | 1 | 5 | 7 | 8 | 9 | 9 | 2 | 4 | | | 8 | | 9 | | 1 | | 5 | | 6 | | 6 | | 6 | | 6 | | 6 | |
|  | 3 | 4 | 4 | 4 | 5 | 4 | 4 | 4 | 6 | 7 | 0 | 1 | 1 | 2 | 2 | 5 | 8 | 0 | 8 | 0 | 7 | 8 | 2 | 8 | | | 9 | | 9 | | 9 | | 6 | | 4 | | 4 | | 5 | | 6 | | 7 | |
|  | 5 | 2 | 5 | 8 | 5 | 3 | 4 | 9 | 0 | 6 | 3 | 2 | 2 | 5 | 6 | 7 | 9 | 5 | 0 | 2 | 6 | 9 | 3 | 2 | | | 1 | | 0 | | 8 | | 5 | | 2 | | 4 | | 9 | | 0 | | 9 | |
| ***Ledebouriella seseloides*** | C | G | C | C | G | C | A | A | T | C | C | C | A | A | A | C | T | **C** | T | C | T | G | C | **T/C^b^** | | | **C** | | C | | **G** | | C | | G | | **T** | | G | | C | | T | |
| ***Peucedanum japonicum*** | C | **A** | C | **T** | G | C | A | A | **C** | **T** | C | **T** | A | **G** | **C** | C | T | T | T | **T** | T | G | C | C | | | A | | C | | A | | C | | G | | C | | **A** | | **T** | | T | |
| ***Glehnia littoralis*** | **T** | G | **G** | C | **A** | **T** | **G** | **T** | T | C | **T** | C | **G** | A | A | **T** | **A** | T | **C** | C | **C** | **A** | **T** | C | | | A | | **T** | | A | | **T** | | **A** | | C | | G | | C | | **C** | |

The bold nucleotides were represented interspecific SNPs distinct from other species. ^a^ Vertical Arabic numerals were meant nucleotide position based on the 45S nrDNA sequence of *L. seseloides* as a reference sequence. ^b^ Heterogeneous nucleotides at the same nucleotide position.

**Table S3.** Cp genome sequence variation of *L. seseloides* between two accessions at the intraspecies level.

| Type of variation | No. | Aligned Position  (bp) | Ls-01 | | Ls-02 | |
| --- | --- | --- | --- | --- | --- | --- |
|  |  |  | **True Position (bp)** | **Sequence** | **True Position (bp)** | **Sequence** |
| **SNP** | 1 | 47,646 | 47,646 | T | 47,644 | G |
|  | 2 | 90,379 | 90,379 | C | 90,377 | T |
|  | 3 | 109,424 | 109,424 | T | 109,389 | G |
| **Indel** | 1 | 9,850 | 9,850 | ATT | 9,850 | AAA |
|  | 2 | 16,405 | 16,405 | TA | 16,405 | T |
|  | 3 | 33,320 | 33,320 | TA | 33,319 | T |
|  | 4 | 91,460 | 91,460 | TGATATTGATGCTAGTGAC | 91,458 | T |
|  | 5 | 94,215 | 94,215 | ATGTAATGTACTT | 94,195 | A |
|  | 6 | 104,476 | 104,476 | AG | 104,442 | A |

**Table S4.** Cp genome sequence variation of *G. littoralis* between two accessions at the intraspecies level.

| Type of variation | No. | Aligned Position (bp) | Gl-01 | | Gl-02 | |
| --- | --- | --- | --- | --- | --- | --- |
|  |  |  | **True Position (bp)** | **Sequence** | **True Position (bp)** | **Sequence** |
| **SNP** | 1 | 99,368 | 99,365 | T | 99,368 | A |
| **Indel** | 1 | 9,750 | 9,750 | A | 9,750 | AG |
|  | 2 | 46,499 | 46,498 | C | 46,499 | CA |
|  | 3 | 85,682 | 85,680 | A | 85,682 | AT |
|  | 4 | 104,599 | 104,596 | AG | 104,599 | A |
|  | 5 | 127,711 | 127,708 | C | 127,710 | CTCTTACTAT |
